# Expansion of T regulatory lymphocytes by murine bone marrow dendritic cells previously stimulated with *Anisakis simplex* larval antigens

**DOI:** 10.1590/0074-02760200560

**Published:** 2021-02-05

**Authors:** Vega Zamora, Marta Rodero, Alexandra Ibáñez-Escribano, Juan C Andreu-Ballester, Susana Mendez, Carmen Cuéllar

**Affiliations:** 1 Universidad Complutense, Facultad de Farmacia, Departamento de Microbiología y Parasitología, Madrid, Spain; 2 Hospital Arnau de Vilanova, Departamento de Investigación, Valencia, Spain; 3 National Institutes of Health, National Institute of Allergy and Infectious Diseases, Division of Microbiology and Infectious Diseases, Respiratory Disease Branch, Rockville, MD, EUA

**Keywords:** Anisakis simplex, mice, tolerogenic dendritic cells, T regulatory cells, IL-10, mitochondrial activity

## Abstract

**BACKGROUND:**

*Anisakis simplex* antigens present immunomodulatory properties by the induction of tolerogenic dendritic cells (DCs) in mice.

**OBJECTIVES:**

To study the capacity of DCs stimulated with *A. simplex* excretory-secretory (ES) or crude extract (CE) to generate T_regs_. To investigate *in vitro* effects of antigens on the metabolic activity of splenocytes induced by LPS or CpG.

**METHODS:**

Phenotypic and functional characterization of T cells co-cultured with *A. simplex*-pulsed DCs was performed by flow cytometry. Lymphocyte mitochondrial respiratory activity was estimated by the Alamar Blue^®^ Assay.

**FINDINGS:**

In C57BL/6J, CD4^+^CD25^-^Foxp3^+^ and CD8^+^CD25^-^Foxp3^+^ populations increased by CE-stimulated-DCs. In BALB/c, CE-stimulated-DCs caused the expansion of CD4^+^CD25^+^Foxp3^+^IL-10+ and CD8^+^CD25^+^Foxp3^+^IL-10+. IFN-γ expression raised in BALB/c CD4^+^CD25^+^ and CD4^+^CD25^-^ for CE and ES, respectively. ES-stimulated-DCs increased CD4^+^CD25^+^ Foxp3^+^ and CD8^+^CD25^-^ Foxp3^+^ expression in T cells. The association of ES or CE with LPS produced the increase in splenocyte activity in C57BL/6J. The association of CE with CpG decreased the proliferation caused by CpG in C57BL/6J.

**MAIN CONCLUSIONS:**

*A. simplex* increase the frequency of T_regs_, which in turn produce IL-10 and IFN-γ. The host genetic base is essential in the development of anti-*Anisakis* immune responses (Th2, Th1, T_reg_).

In a previous work, immunomodulatory properties of *Anisakis simplex* larval antigens were demonstrated by the induction of tolerogenic dendritic cells (DCs) in two strains of mice (C57BL/6J and BALB/c).^1^ DC’s antigen-presenting ability was determined by measuring the expression of membrane markers (MHC I and MHC II, CD80, CD86) and intracellular expression levels of IL-10 and IL-12 cytokines. It was also analyzed whether stimulation with *A. simplex* larval antigens was enhanced by the co-administration of TLR4 and TLR9 agonists [LPS *Escherichia coli* 026B6 and CpG (ODN1826), respectively]. Two differential types of responses were found in the mouse strains studied: C57BL/6J mice developed a more discrete and resistant response, whereas the BALB/c strain showed an acute inflammatory response. These results demonstrate the coexistence of two opposing responses generated by *A. simplex* larval antigens (Th1 *versus* Th2), and confirmed that the host genetic basis plays a role in the development of a Th2 or a T regulatory (T_reg_) response.

Larval products of *A. simplex* slightly increased the proinflammatory cytokine IL-12 characteristic of the Th1 effector response and the suppressive cytokine IL-10 characteristic of the Th2 effector response *in vitro*. Several studies have shown that the expression of IL-10 and IL-12 are closely related in such a way that the production of IL-10 and IL-12 by DCs presents a close reciprocal regulation.^2^ This indicates a possible immunomodulatory mechanism by *A. simplex* larval antigens through these two cytokines, establishing a balance between a Th1 and the Th2 response. Infections with other parasites, like *Schistosoma* spp. or *Trichinella spiralis*, have shown the same balance.^3^ ^,^ ^4^ Several studies have demonstrated that the simultaneous development of Th1 and Th2 anthelminthic immune responses not only limits microbial invasion during the barrier breach, but also promotes tissue repair/regeneration.^5^ In addition, Allen and Wynn^6^ proposed that Th2 immunity evolved to rapidly repair tissue damage caused by helminth parasites instead of controlling them.

Definitely, there are many potential mechanisms of helminth-induced modulation. They include helminth-derived products that act on DCs and Treg cell-inducing helminth-derived molecules have been described.^7^

In light of these findings, we evaluated the capacity of DCs stimulated with *A. simplex* larval products to generate T_regs_. In addition, we investigated the *in vitro* effect of larval antigens of *A. simplex* on the metabolic activity of mouse splenocytes induced by LPS _*E. coli 026B6*_ or CpG (ODN1826).

## MATERIALS AND METHODS

*Animals* - Female C57BL/6J and BALB/c mice were purchased from Charles River Laboratories (L’Arbresle, France). The authors have involved the minimum number of animals to produce statistically reproducible results. All procedures were carried out in accordance with Royal Decree 53/2013, of 1 February, which establishes the basic rules applicable for the protection of animals used in experimentation and other scientific purposes. The project was approved by the Ethics Committee of the Complutense University of Madrid and evaluated and approved by an authorized body authorized by the Community of Madrid for the evaluation of Projects.

*In vitro bone marrow-derived dendritic cells stimulation assays* - Bone marrow-derived DCs (BMDDCs) were cultured in the presence of 20 ng/mL GM-CSF (PeproTech, Rocky Hill, NJ) and collected six days after culture. DCs were then plated in 6-well plates (10^6^/well) before *A. simplex* antigens (excretory-secretory/ES or crude extract/CE) were added to the wells. Antigens were prepared as previously described.^8^ Different stimuli were employed: ES (0.012 µg/mL) or CE (50 µg/mL) for 24 hours (CO_2_ 5%, 37ºC).^1^ A control of DCs cultured in medium without antigens was included in all the experiments. All experiments were performed in triplicate wells for each condition and repeated at least three times. We used the Pierce LAL Chromogenic Endotoxin Quantitation Kit (Thermo Fisher Scientific, Rockford, IL) for the detection of gram-negative bacterial endotoxins. The amounts of endotoxin present in our antigenic samples were negligible compared to the doses of 1 μg/mL of LPS used as positive control.

*Phenotypic and functional characterization of T cells co-cultured with* Anisakis simplex *antigens-pulsed bone marrow-derived dendritic cells* - Splenocytes were purified from spleens of *naïve* C57BL/6J or BALB/c mice by mechanical disruption. Prior to co-culture, red blood cells were lysed for 10 minutes with cold Red Blood Cell Lysing Buffer (SIGMA-ALDRICH). Purified cells were added to cultures containing BMDDCs (five splenocytes: one DC ratio) that have been left unstimulated or had been treated with *A. simplex* antigens. Thirty-six hours after initiation of the co-culture, brefeldin A (*Penicilium brefeldianum*, Life Technologies) (10 µg/mL) was added for 6 h; cells were then collected and fixed in 4% paraformaldehyde. Prior to staining, cells were incubated with an anti-Fcγ III/II receptor and 10% normal mouse serum in phosphate buffered saline (PBS) containing 0.1% bovine serum albumin (BSA), 0.01% NaN_3_. Cells were permeabilized with saponin and stained for the surface markers CD4 [PerCP Rat Anti-Mouse CD4 (BD-Pharmingen)], CD8 [PerCP Rat Anti-Mouse CD8a (BD-Pharmingen)], CD25 [Anti-Mouse CD25 APC (eBioscience)]; for the cytokines IL-12 [Anti-Mouse IL-12/IL-23p40 PE (eBioscience)], IL-10 [PE Rat Anti-Mouse IL-10 (BD Biosciences)], IFN-γ [Anti-Mouse IFN gamma PE (eBioscience)], and the transcription factor Foxp3 [Anti-Mouse/Rat Foxp3 Staining Set PE (eBioscience)]. We carried out incubations of antibodies against surface molecules and intracellular cytokines on ice, for 30 min and 40 min, respectively. Staining of Foxp3 was made following the specific protocol of the manufacturer of the kit. For each sample, at least 20,000 cells were collected using FACSCan/FACSCalibur flow cytometer and CELLQuest software (Becton Dickinson, San Jose, CA) in the Cytometry and Microscopy Center of the Complutense University of Madrid. The data were analysed by Flowing software.

*Splenocyte proliferation assay* - Purified splenocytes from BALB/c and C57BL/6J mice were stimulated with the *A. simplex* antigens as above and/or with the positive controls: LPS _*E. coli 026B6*_ (1 µg/mL) and CpG (ODN1826) (50 µg/mL). All the conditions for the *in vitro* assay were previously standardized. As negative controls splenocytes incubated in RPMI were used. Lymphocyte mitochondrial respiratory activity was estimated by a fluorometric method using the redox dye resazurin. A stock solution (10 mM) of resazurin sodium salt (Sigma-Aldrich) was prepared in PBS and stored at 4ºC protected from light. This assay was carried out using a suspension of splenocytes (7.5 x 10^5^ cells/mL) in RPMI supplemented with 10% (v/v) heat-inactivated serum. Cells were seeded in sterile 96 well microtiter plate (200 µL/well; 150,000 lymphocytes/well) with the different stimuli. After 1, 24 and 48 h of incubation at 37ºC and 5% CO_2_, 20 µL/well of resazurin were added (1 mM). The reduction of resazurin into the fluorescent dye resorufin by the reductase activity of splenocytes was sequentially measured during the culture period in a plate fluorimeter (Infinite 200, TECAN) at λ_excitation_: 535 nm and λ_emission_: 590 nm following the Alamar Blue^®^ Assay (US Patent 5,501,959) recommendations. Experiments were carried out for both mouse strains (BALB/c and C57BL/6J) at least four times. A resazurin control in PBS without cells was included in all the plates. The fluorescence derived from the spontaneous oxidation of the probe was subtracted from the fluorometric results of the test samples. In order to estimate the mitochondrial respiratory activity of each sample stimulation indexes (SI) were calculated for each resazurin incubation interval.

*Statistics* - Data are presented as mean ± standard deviation (SD). Differences were analyzed for significance. Non-parametric tests were performed (Wilcoxon and Monte Carlo Tests) to compare the means. To compare groups of stimuli, Mann-Whitney U Test was used. One-way analysis of variance (ANOVA) followed by the Tukey’s honest significant difference post-hoc test was applied to evaluate differences in SI for the resazurin assay (SPSS 19, Inc., Chicago, IL, USA). A p value less than 0.05 was used as the threshold for significance.

## RESULTS

*Co-incubation of splenic cells with bone marrow-derived dendritic cells pulsed with Anisakis simplex antigens* - The following populations were analyzed: CD4^+^ and CD8^+^ T cells; and the subpopulations CD4^+^CD25^+^, CD8^+^CD25^+^, CD4^+^CD25^-^ and CD8^+^CD25^-^ T cells. In addition, we measured the cell percentages that express IL-10 and IFN-γ. We also determined the expression of the transcription factor Foxp3.

**Fig. 1:**
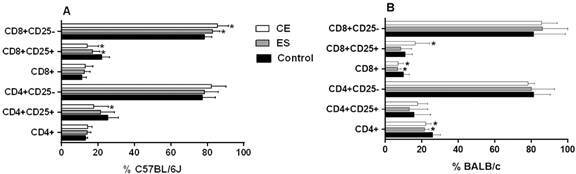
percentages of T cell subsets after 48 h of co-culture of mice splenocytes and bone marrow-derived dendritic cells (BMDDCs) previously stimulated with *Anisakis simplex* larval antigens excretory-secretory (ES) or crude extract (CE). Panel A. C57BL/6J. Panel B. BALB/c. Percentages of total CD4^+^ or CD8^+^, CD4^+^CD25^+^ or CD4^+^CD25^-^ and CD8^+^CD25^+^ or CD8^+^CD25^-^ cells. Data are expressed as mean ± standard deviation (SD) of data from three independent experiments. Asterisk indicates statistically significant differences respect to the control, *p < 0.05.

**Fig. 2:**
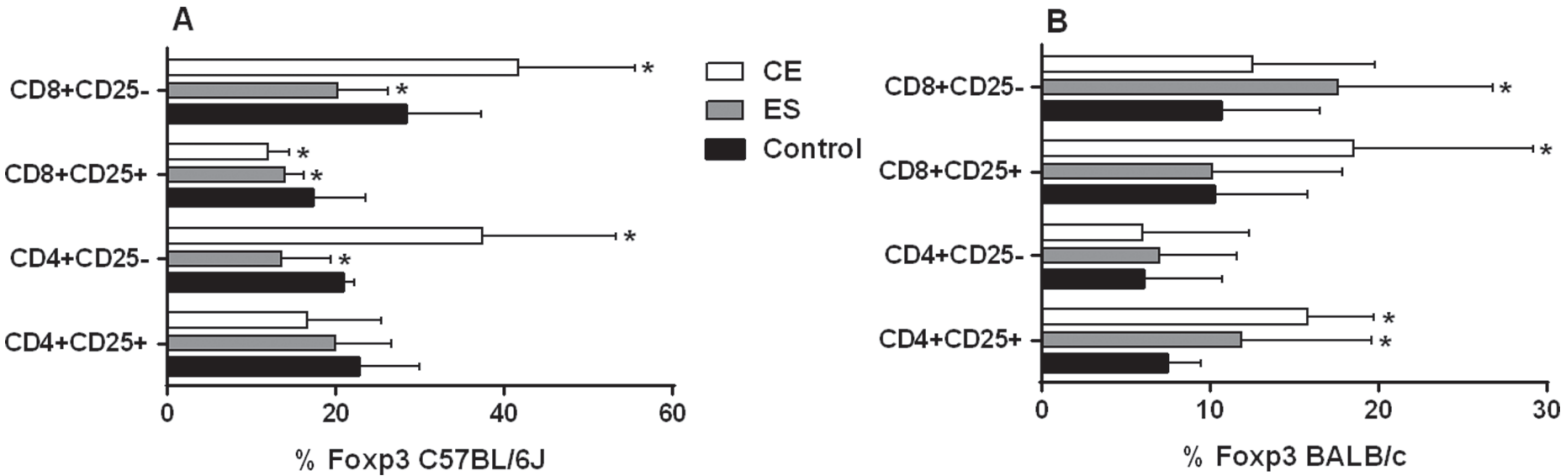
percentages of T cell subsets after 48 h of co-culture of mice splenocytes and bone marrow-derived dendritic cells (BMDDCs) previously stimulated with *Anisakis simplex* larval antigens excretory-secretory (ES) or crude extract (CE). Panel A C57BL/6J. Panel B: BALB/c. Percentages of CD4^+^CD25^+^ or CD4^+^CD25^-^ and CD8^+^CD25^+^ or CD8^+^CD25^-^ that express Foxp3. Data are expressed as mean ± standard deviation (SD) of data from three independent experiments. Asterisk indicates statistically significant differences respect to the control, *p < 0.05.

C57BL/6J BMDDCs previously stimulated with CE caused a significant increase in the frequency of CD8^+^CD25^-^ T cells (p < 0.05) (Fig. 1A). Likewise, the number of CD25^+^ total cells was decreased compared to the negative control (p < 0.05 for CD8^+^CD25^+^ and CD4^+^CD25^+^ T cells) (Fig. 1A). When cells were stimulated with ES products, the percentage of CD8^+^CD25^-^ T cells was increased, while the CD8^+^CD25^+^ T cell subset was diminished (both p < 0.05) (Fig. 1A). Interestingly, C57BL/6J BMDDCs previously stimulated with CE achieved higher rates of CD4^+^CD25^-^Foxp3^+^ cells and CD8^+^CD25^-^Foxp3^+^ cells compared to the negative control, although ES exhibited the opposite response (p < 0.05) (Fig. 2A). Significant differences were found between CE and ES for Foxp3 expression in both CD8^+^CD25^-^ (p < 0.05) and CD4^+^CD25^-^ T cells (p < 0.01) (Fig. 2A).

**Fig. 3:**
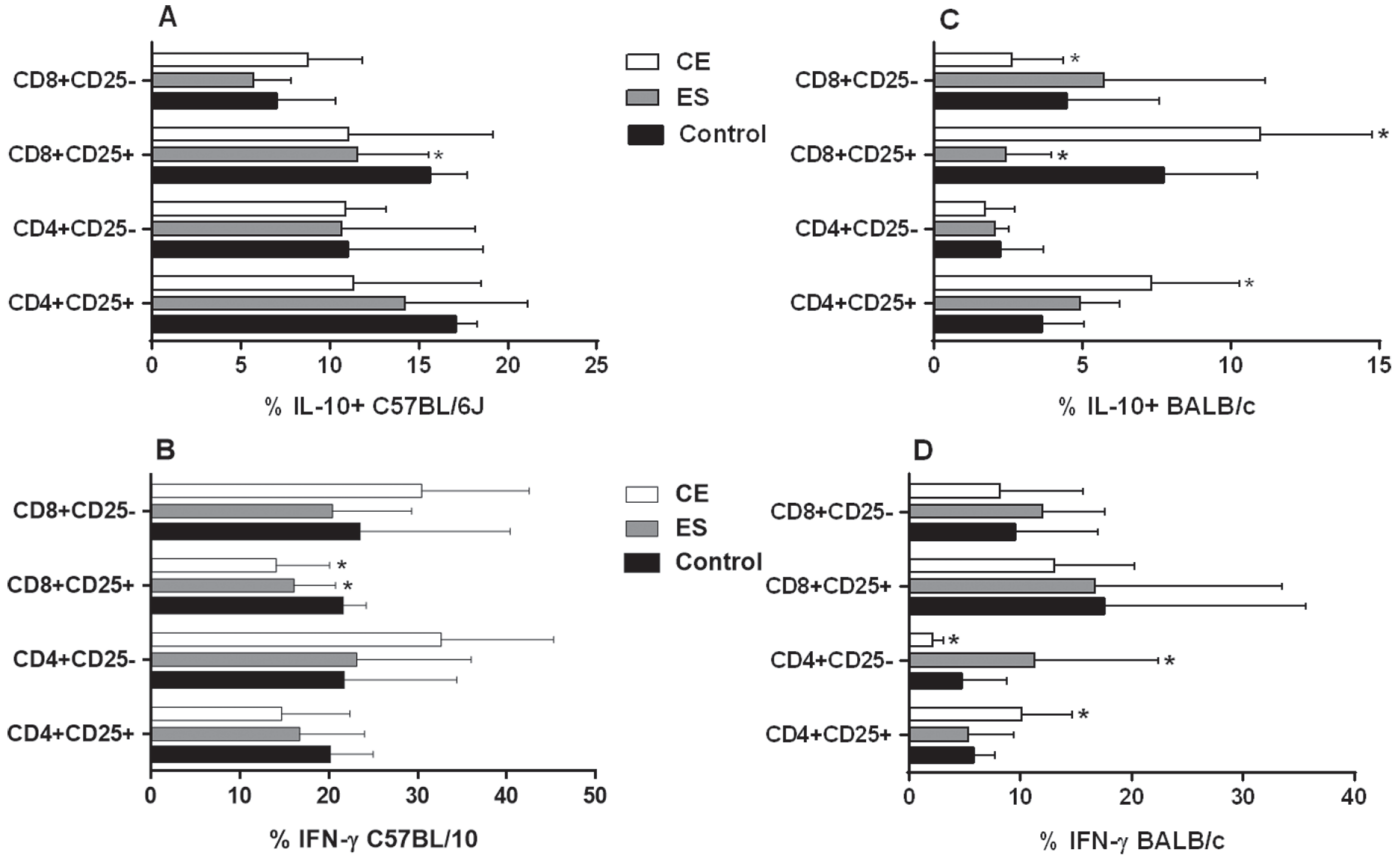
percentages of T cell subsets after 48 h of co-culture of mice splenocytes and bone marrow-derived dendritic cells (BMDDCs) previously stimulated with *Anisakis simplex* larval antigens excretory-secretory (ES) or crude extract (CE). Panels A and B: C57BL/6J. Panels C and D: BALB/c. Percentages of CD4^+^CD25^+^ or CD4^+^CD25^-^ and CD8^+^CD25^+^ or CD8^+^CD25^-^ that express IL-10 or IFN-γ. Data are expressed as mean ± standard deviation (SD) of data from three independent experiments. Asterisk indicates statistically significant differences respect to the control, *p < 0.05.

**Fig. 4:**
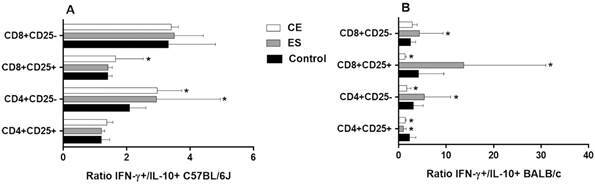
ratio of IFN-γ/IL-10 expression in CD4^+^CD25^+^, CD4^+^CD25^-^, CD8^+^CD25^+^ and CD8^+^CD25^-^ T cell subsets after 48 h of co-culture of mice splenocytes and bone marrow-derived dendritic cells (BMDDCs) previously stimulated with *Anisakis simplex* larval antigens excretory-secretory (ES) or crude extract (CE). Panel A: C57BL/6J. Panel B: BALB/c. Data are expressed as mean ± standard deviation (SD) of data from three independent experiments. Asterisk indicates statistically significant differences respect to the control, *p < 0.05.

Concerning cytokine expression, an inhibitory response was observed in C57BL/6J CD4^+^CD25^+^ and CD8^+^CD25^+^ activated T cells in almost all conditions analyzed when compared to the negative control. This difference was significant in ES-treated CD8^+^CD25^+^IL-10^+^ T cells and in CE and ES-treated CD8^+^CD25^+^IFN-γ^+^ T cells (p < 0.05) (Fig. 3A-B). Finally, BMDDCs previously stimulated with CE promoted the expansion of CD8^+^CD25^-^IL-10^+^ (Fig. 3A), CD4^+^CD25^-^IFN-γ^+^ and CD8^+^CD25^-^IFN-γ^+^ T cells with a trend towards significance (Fig. 3B). This trend was confirmed in the case of CD4^+^CD25^-^ T cells, which showed a significant increase of the ratio IFN-γ^+^/IL-10^+^ expression after CE and ES/BMDDC stimulation (p < 0.05) (Fig. 4A).

When experiments were carried out using BALB/c cells, stimulation of BMDDCs with ES or CE caused a significant decreased of percentages of total CD4^+^and CD8^+^ T cells compared to the negative control (p < 0.05) (Fig. 1B). Also, the percentages of CD4^+^CD25^+^Foxp3^+^ and CD8^+^CD25^+^Foxp3^+^ T_regs_ were significantly increased by BALB/c BMDDCs stimulated with CE; ES, however, promoted Foxp3 expression by both CD4^+^CD25^+^ and CD8^+^CD25^-^ T cell subsets (p < 0.05) (Fig. 2B). Moreover, CE and ES significantly increased CD4^+^CD25^+^IFN-γ^+^ and CD4^+^CD25^-^IFN-γ^+^ T cells, respectively (p < 0.05) (Fig. 3D). However, the expression of CD8^+^CD25^+^IFN-γ^+^ T cells was inhibited by CE antigens with a trend towards significance compared to the negative control (Fig. 3D), as demonstrated by the significant decrease of the ratio IFN-γ^+^/IL-10^+^ presented in the Fig. 4B (p < 0.05). The same trend was observed in the CD4^+^CD25^+^ T cell subset, where stimulation of BMDDCs with CE and ES induced a significantly reduction of the ratio of IFN-γ^+^/IL-10^+^ T cells (p < 0.05) (Fig. 4B). Moreover, CE exposure of BALB/c BMDDCs boosted the proliferation of CD4^+^CD25^+^IL-10^+^ as well as of CD8^+^CD25^+^IL-10^+^ T cell subsets (p < 0.05) (Fig. 3C). Subsequently, the ratio of IFN-γ^+^/IL-10^+^ T cells was significantly reduced in both T_reg_ populations (p < 0.05) (Fig. 4B). On the other hand, ES stimulation of BALB/c BMDDCs not only lead to a significant decrease of CD8^+^CD25^+^IL-10^+^ T cells compared with CE (p < 0.01) (Fig. 3C), but also amplified the ratio of IFN-γ^+^/IL-10^+^ T cells in this cell subset as well as in the CD8^+^CD25^-^ T cell population (p < 0.05) (Fig. 4B).

**Fig. 5:**
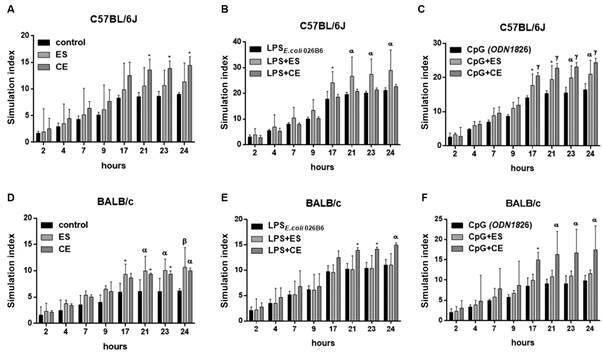
stimulation index (SI) of C57BL/6J or BALB/c mouse splenocytes after 1 h of stimulation with *Anisakis simplex* ES or CE or medium alone (Panels A and D), with LPS _*E. coli 026B6*_ alone or associated with *A. simplex* excretory-secretory (ES) or crude extract (CE) (Panels B and E) or with CpG (ODN1826) alone or associated with *A. simplex* ES or CE; and then incubated for 24 h with resazurin. Data are expressed as mean ± standard deviation (SD) of data from four independent experiments. Statistically significant differences with respect to the control, LPS _*E. coli 026B6*_ or CpG (ODN1826), *p < 0.05, ^α^p < 0.01, ^β^p < 0.001 or ^γ^p < 0.0001, are indicated. C57BL/6J cells incubated with medium alone showed significant increase of SI at 17 h (p < 0.05) 21 h (p < 0.05), 23 h and 24 (p < 0.01). C57BL/6J cells incubated with LPS _*E. coli 026B6*_ alone showed significant increase of SI at 17 h (p < 0.0001), 21 h, 23 h and 24 h (p < 0.0001). C57BL/6J cells incubated with CpG (ODN1826) alone showed significant increase of SI at 9 h (p < 0.01), 17 h, 21 h, 23 h and 24 h (p < 0.001). BALB/c cells incubated with medium alone showed significant increase of SI at 17 (p < 0.01) 21 h, 23 h and 24 h (p < 0.001). BALB/c cells incubated with LPS _*E. coli 026B6*_ alone showed significant increase of SI at 9 h (p < 0.05), 17 h, 21 h, 23 h and 24 h (p < 0.0001). BALB/c cells incubated with CpG (ODN1826) alone showed significant increase of SI at 21 h (p < 0.05), 23 h, 24 h (p < 0.05).

*Splenocyte proliferation assay* - SI of C57BL/6J or BALB/c mouse splenocytes were calculated following 1 h incubation with ES or CE or medium alone and in combination with LPS and CpG. Resazurin was then added for 24 h. Control C57BL/6J and BALB/c cells incubated with medium, LPS or CpG showed significant increase in SI (Fig. 5). In C57BL/6J, excretory-secretory (ES) or crude extract (CE) increased SIs when compared to the negative control of medium. These differences were significant in the case of CE stimulation (Fig. 5A). If C57BL/6J splenocytes were co-stimulated with ES/CE and LPS, we observed that ES addition significantly increased SI achieved by LPS alone (Fig. 5B). Similarly, co-incubation of CpG with ES/CE significantly augmented SIs achieved by CpG alone (Fig. 5C). In BALB/c, ES/CE increased SIs in a significant manner compared to control cells or medium (Fig. 5D). Co-incubation with CE significantly increased SIs produced by LPS alone (Fig. 5E). As before, addition of CpG to CE produced a significant increase of SIs compared to CpG alone (Fig. 5F).

**Fig. 6:**
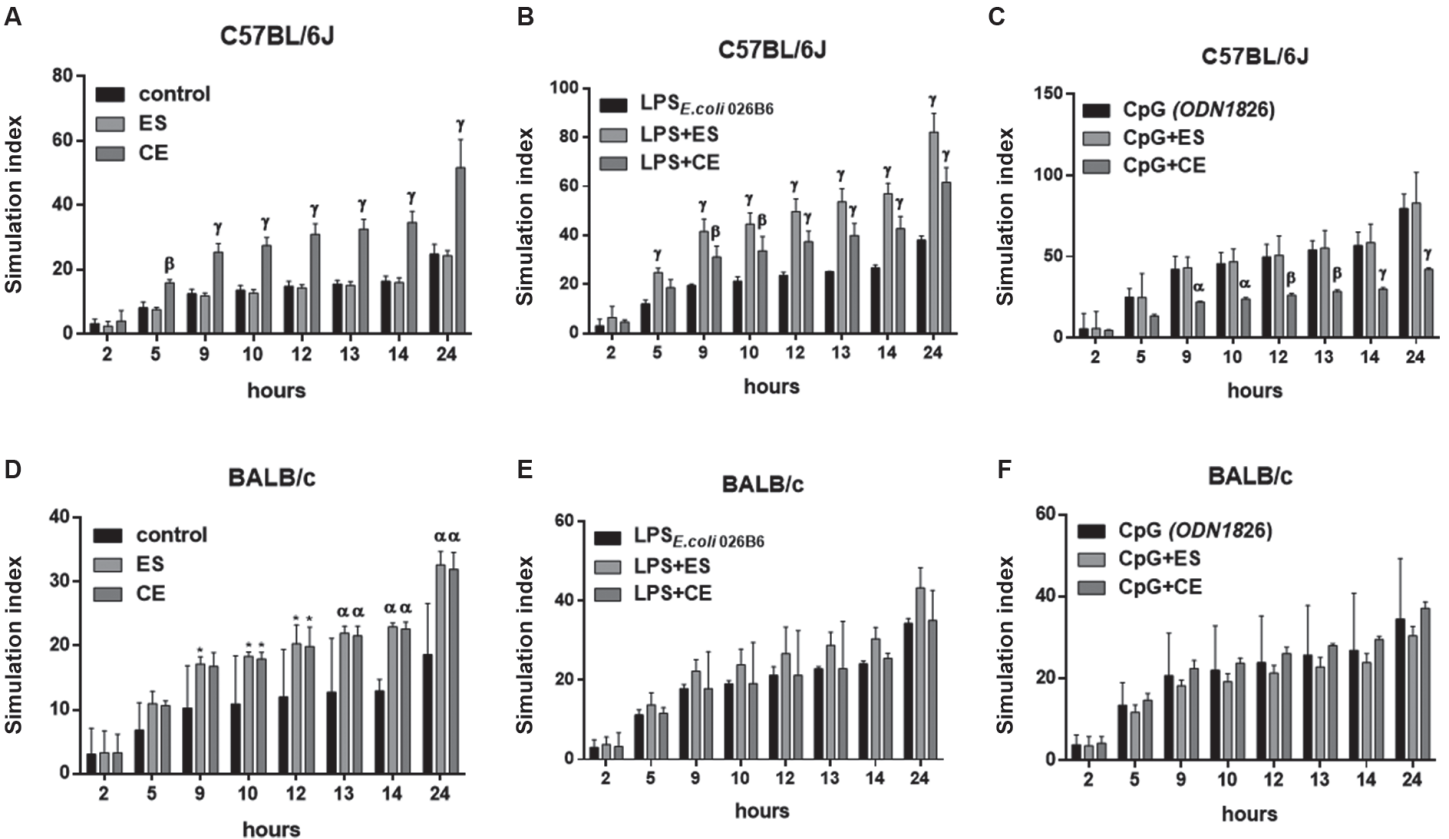
stimulation index (SI) of C57BL/6J or BALB/c mouse splenocytes after 24 h of stimulation with *Anisakis simplex* excretory-secretory (ES) or crude extract (CE) or medium alone (Panels A and D), with LPS _*E. coli 026B6*_ alone or associated with *A. simplex* ES or CE (Panels B and E) or with CpG (ODN1826) alone or associated with *A. simplex* ES or CE; and then incubated for 24 h with resazurin. Data are expressed as mean ± standard deviation (SD) of data from four independent experiments. Statistically significant differences with respect to the control, LPS _*E. coli 026B6*_ or CpG (ODN1826), *p < 0.05, ^α^p < 0.01, ^β^p < 0.001 or ^γ^p < 0.0001, are indicated. C57BL/6J cells incubated with medium alone showed significant increase of SI at 9 h (p < 0.001), 10 h, 12 h, 13 h, 14 h and 24 h (p < 0.0001). C57BL/6J cells incubated with LPS _*E. coli 026B6*_ alone showed significant increase of SI at 9 h (p < 0.0001), 10 h, 12 h, 13 h, 14 h and 24 h (p < 0.0001). C57BL/6J cells incubated with CpG (ODN1826) alone showed significant increase of SI at 5 h (p < 0.05), 9 h, 10 h, 12 h, 13 h, 14 h and 24 h (p < 0.0001). BALB/c cells incubated with medium alone showed significant increase of SI at 12 h (p < 0.05), 13 h, 14 h (p < 0.05), 24 h (p < 0.0001). BALB/c cells incubated with LPS _*E. coli 026B6*_ alone showed significant increase of SI 9 h (p < 0.01), 10 h (p < 0.01), 12 h (p < 0.001), 13 h, 14 h and 24 h (p < 0.0001). BALB/c cells incubated with CpG (ODN1826) alone showed significant increase of SI at 9 h (p < 0.01), 10 h (p < 0.01), 12 h, 13 h (p < 0.001), 14 h and 24 h (p < 0.0001).

SIs were calculated following 24 h incubation with antigens and controls with the same conditions described above. Control C57BL/6J and BALB/c cells incubated with medium, LPS or CpG showed significant increase of SIs (Fig. 6). In C57BL/6J, addition of CE significantly increased SIs compared to medium (Fig. 6A). Co-stimulation of C57BL/6J splenocytes with ES/CE and LPS significantly increased SIs compared to incubation with LPS alone (Fig. 6B). Interestingly, the addition of CpG to CE significantly inhibited SIs induced by CpG alone (Fig. 6C). In BALB/c, both larval antigens increased SIs in a significant manner compared to medium (Fig. 6D). No effects were observed when ES/CE were combined either with LPS or with CpG (Fig. 6E-F).

**Fig. 7:**
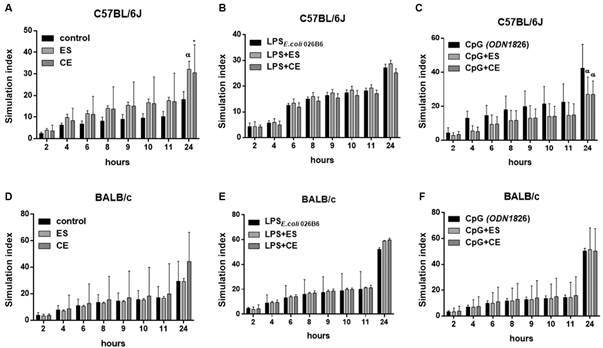
stimulation index (SI) of C57BL/6J or BALB/c mouse splenocytes after 48 h of stimulation with *Anisakis simplex* excretory-secretory (ES) or crude extract (CE) or medium alone (Panels A and D), with LPS _*E. coli 026B6*_ alone or associated with *A. simplex* ES or CE (Panels B and E) or with CpG (ODN1826) alone or associated with *A. simplex* ES or CE; and then incubated for 24 h with resazurin. Data are expressed as mean ± standard deviation (SD) of data from four independent experiments. Statistically significant differences with respect to the control, LPS _*E. coli 026B6*_ or CpG (ODN1826), *p < 0.05, ^α^p < 0.01, ^β^p < 0.001 or ^γ^p < 0.0001, are indicated. C57BL/6J cells incubated with medium alone showed significant increase of SI at 24 h (p < 0.05). C57BL/6J cells incubated with LPS _*E. coli 026B6*_ alone showed significant increase of SI at 6 h (p < 0.0001), 8 h, 9 h, 10 h, 11 h and 24 h (p < 0.0001). C57BL/6J cells incubated with CpG (ODN1826) alone showed significant increase of SI at 10 h (p < 0.05), 11 h (p < 0.05) and 24 h (p < 0.0001). BALB/c cells incubated with medium alone showed significant increase of SI at 24 h (p < 0.05). BALB/c cells incubated with LPS _*E. coli 026B6*_ alone showed significant increase of SI at 10 h (p < 0.05), 11 h (p < 0.05) and 24 h (p < 0.0001). BALB/c cells incubated with CpG (ODN1826) alone showed significant increase of SI at 24 h (p < 0.0001).

SIs of splenocytes were calculated following 48 h incubation with antigens or medium alone and in combination with LPS and CpG. Control C57BL/6J and BALB/c cells incubated with medium, LPS or CpG showed significant increase of SIs (Fig. 7). In the case of C57BL/6J, ES/CE significantly increased SIs compared to the negative control of medium (Fig. 7A). No effects were observed when ES/CE were combined with LPS (Fig. 7B). On the contrary, both antigens produced a significant decrease in the SI when they associated with CpG (Fig. 7C). None of the two antigens studied produced any change in the SIs produced by their respective controls in BALB/c (Fig. 7D-F).

## DISCUSSION

The evaluation of DC activation can be determined by measuring expression of surface molecules and production of cytokines, but it can also be established by their capability to present antigens to T cells that leads to their activation and differentiation. In our previous work, we demonstrated that even though murine DCs stimulated *in vitro* with *A. simplex* larval antigens did not reach a complete activated phenotype, they showed ability to present antigens to T cells and cause polarization.^1^ Likewise, Napoletano et al. observed that *Anisakis pegreffii* generated human DCs with an impaired phenotype as demonstrated by low expression of HLAII- DR and CD86 costimulatory molecules.^9^

In this work, co-cultivation of mouse splenocytes with DCs previously stimulated with *A. simplex* larval antigens caused the expansion of T_regs_. The frequencies of CD4^+^CD25^-^Foxp3^+^ and CD8^+^CD25^-^Foxp3^+^ T cell populations were increased in C57BL/6J mice by DCs stimulated with CE, but percentages decreased after incubation with ES. In addition, DCs stimulation with CE decreased the frequency of CD8^+^CD25^+^ T cells. Conversely, CE stimulation significantly enhanced the expression of CD8^+^CD25^-^ T cells compared to the negative control. Therefore, as with BALB/c mice, tolerogenic DCs promoted by *A. simplex* larval antigens appeared to cause T_reg_ expansion.

The study main limitations are a small sample plus high variable standard deviations. Thus, it is difficult to come up with consistent conclusions from the results. However, it is also important to remember this is an *in vitro* study, and the goal was to obtain preliminary data that could open the way to the design of future *in vivo* experiments. Our results may lack homogeneity, but it confirms the dual activity that *A. simplex* has over the immune system of the host, and it is the first study that shows *A. simplex* larval antigens are able to regulate the close interaction between DCs and lymphocytes.

IFN-γ expression was raised mainly in CD4^+^CD25^-^ and CD8^+^CD25^-^ T cells after incubation with CE in C57BL/6J mice, and in CD4^+^CD25^+^ T cells stimulated with CE and CD4^+^CD25^-^ T cells stimulated with ES in BALB/c mice. Likewise, oral administration of *Anisakis typica* CE increased significantly CD4+IFN-γ+ cells in BALB/c mice.^10^ This differs with the findings of the study of *Heligmosomoides polygyrus*, where CD4^+^CD25^+^ T cells did not express IFN-γ.^11^ However, *in vivo* experiments carried out in mice using the *Ascaris suum* antigen PAS-1 demonstrated that CD8^+^γδ^+^ T cells were able to modulate airway allergic inflammation through the production of IFN-γ.^12^ These results resemble the duality of the immune responses caused by *A. simplex* larval antigens described by us in this work. *Ascaris* and *Anisakis* are nematodes that belong to the same Order (*Ascaridida*); their ability to originate allergic responses in their hosts,^13^ where T_reg_ populations develop and IFN-γ is produced, has been well established. Studies demonstrating that IFN-γ possesses immune regulatory ability and is implicated in allergic responses are becoming more abundant.^14^ Recently, Jung et al.^15^ demonstrated the key role of IFN-γ in the induction of colitis in mice after intratracheal ovalbumin exposure. In addition, Chen and Liu^16^ demonstrated the immunoregulatory ability of CD4^+^IL-10^+^IFN-γ^+^ T cells, as well as their implication in chronic infections. IL-10 expression would prevent an excessive acute response from the host, inhibiting the production of IL-12 and, concomitantly, the development of a Th2 response. Besides, IFN-γ could increase a Th1 response and production of IL-27 by antigen presenting cells (APCs). Finally, APCs could activate CD4^+^IL-10^+^IFN-γ^+^ T cells. Despite this evidence, there is great controversy about the phenotype and function of regulatory populations, so it is difficult to draw strong conclusions based on the literature. For example, *H. polygyrus* treated DCs were able to generate *in vitro* CD4^+^CD25^+^ T cells that secrete IL-10 but did not express Foxp3.^11^ On the other hand, Wang et al.^17^ showed that *Echinococcus granulosus* ES-treated DC co-cultures expanded the frequency of CD4^+^CD25^+^Foxp3^+^ T cells whose regulatory role, interpreted as a potential mechanism of immune evasion, was independent of IL-10. Tang et al.^18^ also demonstrated that CD4^+^CD25^+^ T_regs_ play an important role on the *Schistosoma japonicum* immune evasion from the host immune response. In our experimental conditions, BALB/c mice showed a significant decrease of CD4^+^ and CD8^+^ T cells after co-culture with DCs exposed to *A. simplex* antigens. This finding was associated with higher levels of CD4^+^CD25^+^Foxp3^+^ and CD8^+^CD25^+^Foxp3^+^ T_regs_ as well as with higher percentages of IL-10^+^ cells within both CD4^+^CD25^+^ and CD8^+^CD25^+^ T cell subsets, supporting a regulatory phenotype. These outcomes are consistent with the ability of CD4^+^CD25^+^ T_regs_ of suppressing CD4^+^ and CD8^+^ T cells by a direct contact mechanism and by IL-10 secreted by CD4^+^ CD25^+^ T_regs_.^19^

The development of immature and incompletely activated DCs has been described for *H. polygyrus* in similar experimental conditions. In those studies, DCs induced the proliferation of CD4^+^CD25^+^ T cells that produced IL-10 *in vitro*, and inhibited proliferation of IFN-γ producing cells. These data suggested that the ES antigens of *H. polygyrus* were able to inhibit both Th1 and Th2 responses by an increase on a CD4^+^CD25^+^ T cell suppressor population.^11^ In fact, it has been described that T_regs_ are involved in both allergic and autoimmune responses (Th2 and Th1, respectively).^20^ It is noteworthy that *A. simplex* and *H. polygyrus* are intestinal parasites, and that T_regs_ differentiation induced by intestinal DCs exposed to helminths is one of the main mechanisms that lead to immunomodulation.^11^ Junginger et al.^21^ also demonstrated elevated rates of Foxp3^+^ lymphocytes in the intestinal mucosa of nematode-infected dogs. Taken together, the reported results in BALB/c mice, suggest a new mechanism by which *A. simplex* larval antigens are able to immunomodulate the immune system unknown until now; i.e., generation of T_regs_ by immature DCs.

Together, these results suggest that *A. simplex* larval antigens can stimulate tolerogenic DCs that could lead to T_regs_ *in vitro*. Tolerogenic DCs would produce moderate amounts of IL-12 and TNF-α, which would promote the expansion of T cells producers of IFN-γ (Th1 response).

The development of these apparently opposed responses could be related to the two different mouse strain phenotypes: the more susceptible BALB/c and the more resistant C57BL/6J. As it was previously reported, *A. simplex* antigens promoted both immature and inflammatory DCs with a higher percentage of CD11c^+^IL-10^+^ cells in C57BL/6J mice at 24 h of stimulation with larval antigens.^1^ Furthermore, the expression of cytokines that exert opposing effects, could be consider as an autoregulatory mechanism: IL-10-producing T cell populations could inhibit initial Th1 and Th2 responses, while IFN-γ-producing T cell populations could promote a Th1 response but at the same time stimulate the production of IL-10 in order to avoid an excess of inflammation. Providing that the production of IL-10 and IFN-γ is in harmony, Th1 and Th2 responses would stay apart, and a regulatory phenotype would prevail.

Murine immune responses to *A. simplex* infection are complex and entail Th2 predominance over Th1 at the beginning.^8^ Similar results have been described in humans.^22^ According to our study, *A. simplex* infection could be accompanied by tolerogenic DCs activation, leading to T_reg_ populations which could produce IL-10 and IFN-γ. Although there are not many studies regarding the effect of *A. simplex* larval antigens on cell proliferation, Raybourne et al.^23^ reported that *A. simplex* ES larval antigens were able to inhibit lymphoproliferation in cells activated by Concanavalin A and bacterial LPS.

Resazurin-based proliferation assays are fast and reproducible methods to quantify lymphocyte mitochondrial activity that appear to accurately reflect cell numbers in a linear fashion and can be continuously monitored over time.^24^ In our study, we investigated the effect of *A. simplex* larval antigens alone or with the mitogens LPS/CpG in *naïve* murine splenocytes compared to a negative control and the mitogens alone. We also compared BALB/c and C57BL/6J responses. In both strains, ES and especially CE appear to stimulate the mitochondrial respiratory activity respect to the negative control. This could be explained *in vivo* due to granuloma formation. ES antigens released by living larvae induce a neutrophilic infiltration in an early stage, which is then replaced by eosinophils as the granuloma matures and larvae are killed. In contrast, CE antigens released by parasite damage exhibit a potent chemotactic activity for eosinophils, inducing intense eosinophilic phlegmonous reactions.^25^ Therefore, ES could potentially skew the immune response towards a Th2 response, while CE could be causing Th1 polarization.

On the other hand, the association of ES or CE with LPS produced different responses after 24 h of stimulation: in BALB/c mice, neither ES nor CE modified the mitochondrial respiratory activity caused by LPS alone (already higher than the negative control of medium), while in C57BL/6J mice, ES and CE increased the effect of LPS activation. The fact that the addition of *A. simplex* antigens did not show any effects on the response of LPS in BALB/c mice but did in C57BL/6J mice clearly demonstrates the divergent responses of both strains. Previous studies have shown that the monocyte/macrophage LPS response is slightly regulated by IL-10 and IL-12.^26^ BALB/c mice are highly reactive to *A. simplex* antigens, generating an acute inflammatory response, with higher expression of IL-12 and lower expression of IL-10 than C57BL/6J mice.^1^ Interestingly, TLR4 expression is higher in BALB/c compared to C57BL/6.^27^

Conversely, co-incubation of CE with CpG clearly decreased the mitochondrial respiratory activity caused by CpG alone in C57BL/6J mice, a strain that expresses higher levels of TLR9 compared to BALB/c.^27^ This inhibitory effect was also observed after 48 h of stimulation with ES/CE, while no change was observed in BALB/c. IL-10 has several functions, including inhibit cellular proliferation.^28^ Thus, it is not surprising that C57BL/6J mice, which released higher levels of IL-10 (compared to BALB/c), showed a decrease in the mitochondrial respiratory activity caused by CpG alone. Likewise, BALB/c and C57BL/6, display dramatically different mucosal immune responses.^29^ Therefore, the immune response developed to *A. simplex* will not only depend on the own regulatory mechanism of the parasite, but also of the host genetic phenotype.^30^ BALB/c mice would match with the helminth infection resistant phenotype, characterized by acute inflammatory reactions and a Th2 response common in endemic helminth areas, while C57BL/6J mice would represent the non-resistant phenotype, with the infection development and more moderate/chronic infection.^22^

Similar results have been described in other parasites like *H. polygyrus*. This parasite presents a Th2 response accompanied with an activation of T_regs_ and Th1 cytokine inhibition.^7^ *Necator americanus* is also well-known for inhibit cellular proliferation.^31^ However, the mechanisms involved in supressing T lymphocyte proliferation are not clear yet, but include IL-10 production, release of IFN-γ by NK cells,^32^ direct action of parasite antigens^31^ and reduced expression of TLRs.^33^ The results of this study agree to other studies in other helminths like *H. polygyrus* or *N. americanus*, which also seem to inhibit T cell proliferation. Even though *A. simplex* larval antigens not always caused an inhibition of proliferation and this inhibition is strain dependent, our results suggest that *A. simplex* may exert regulatory. Our work also confirms that hosts genetics are essential for the development of Th2 or T_regs_ cells.

In conclusion, incubation with ES and CE larval antigens of *A. simplex* induce tolerogenic DCs that increase the frequency of regulatory T lymphocytes *in vitro*, which in turn produce IL-10 and IFN-γ. These cytokines could supress the development of Th1 and/or Th2 responses at the beginning of the infection. Moreover, the effects observed of the *A. simplex* larval antigens on the lymphoproliferation assays suggest unknown regulatory mechanisms of this parasite and confirm that the host genetic base is essential in the development of one immune response predominant over the others (Th2, Th1, T_reg_). Thus, differences in the reactivities of lymphocytes to ES/CE antigens through TLRs may underlie resistance and susceptibility of human patients to *A. simplex* infection.
